# ASO Author Reflections: Advances in Axillary Management for Breast Cancer After Neoadjuvant Chemotherapy

**DOI:** 10.1245/s10434-025-17340-7

**Published:** 2025-04-20

**Authors:** Marissa K. Boyle, Armando E. Giuliano

**Affiliations:** Division of Surgical Oncology, Department of Surgery, Cedars-Sinai Medical Center, Los Angeles, CA USA

## Past

Neoadjuvant chemotherapy (NAC) is used to downstage the axilla for patients with node-positive breast cancer. Multiple randomized clinical trials showed an acceptable false negative rate (FNR) predictive of the axilla after NAC when three or more sentinel lymph nodes were excised using dual mapping, leading to fewer axillary lymph node dissections for patients with tumor-free sentinel nodes.^1–4^ The biopsy-proven clipped node was not a sentinel node in 23% of patients, leading to the development of techniques to target and excise the clipped node.^5^

## Present

Targeted axillary dissection (TAD) was developed to retrieve the biopsy-proven metastasis.^5^ While the technique has limitations and can be challenging to reproduce without a skilled radiologist to target the node, placement of a localization device in the clipped node before NAC improves accuracy. Using TAD, selective removal of the clipped node demonstrated a low FNR. Although it is encouraging to retrieve the clipped node, either method of TAD versus sentinel lymph node biopsy alone leads to excellent staging and regional control with low axillary recurrence.^6^

## Future

With advances in systemic treatment, de-escalation trials for breast cancer patients with response to NAC and axillary pathologic complete response continue to evolve. Results of the NRG Oncology/NSABP B-51/RTOG 1304 trial evaluating invasive breast cancer recurrence in this population of patients with positive axillary nodes who are ypN0 after NAC may lead to omission of regional nodal radiotherapy, and excision of the clipped node may help guide radiation oncologists in their decision for de-escalation.^7^ For patients with residual tumor-involved sentinel lymph nodes after NAC, Alliance A11202 will guide the decision for axillary radiation as an alternative to axillary lymph node dissection for local control of axillary disease.^8^
